# Cofilin 1 activation prevents the defects in axon elongation and guidance induced by extracellular alpha-synuclein

**DOI:** 10.1038/srep16524

**Published:** 2015-11-12

**Authors:** Sharada Tilve, Francesco Difato, Evelina Chieregatti

**Affiliations:** 1 Department of Neuroscience and Brain Technologies, Istituto Italiano di Tecnologia, 16163 Genoa, Italy

## Abstract

Impaired adult neurogenesis and axon traumatic injury participate in the severity of neurodegenerative diseases. Alpha-synuclein, a cytosolic protein involved in Parkinson’s disease, may be released from neurons, suggesting a role for excess secreted alpha-synuclein in the onset and spread of the pathology. Here we provide evidence that long term exposure of young neurons to extracellular alpha-synuclein hampers axon elongation and growth cone turning. We show that actin turnover and the rate of movement of actin waves along the axon are altered, due to alpha-synuclein-induced inactivation of cofilin. Upon laser disruption of microfilaments, healing of axons is favored by the increased phosphorylation of cofilin, however, at later time points; the defect in neurite extension prevails, being lost the regulation of cofilin activity. Importantly, overexpression of the active form of cofilin in neurons exposed to alpha-synuclein is able to restore the movement of actin waves, physiological axon elongation and growth cone turning. Our study reveals the molecular basis of alpha-synuclein-driven deficits in growth and migration of newborn neurons, and in elongation and regeneration of adult neurons.

Rare forms of Parkinson’s disease (PD) resulting from mutations of alpha-synuclein (Syn) or increased expression of wild-type (wt) Syn are characterized by early onset and autosomal-dominant inheritance, implicating Syn in the pathogenesis of the disease^1^,^2^. Syn levels may also have a role in the pathogenesis of sporadic PD; nucleotide polymorphisms highly associated with PD, and affecting Syn levels by altering gene transcription or mRNA stability, were recently identified^3^,^4^. Syn detection in the cerebrospinal fluid and in the plasma^5^ opened the field to the study of non cell-autonomous mechanisms in PD. Healthy dopaminergic grafts implanted in the striatum undergo degeneration accompanied by Syn-containing Lewy bodies, suggesting a potential role of secreted extracellular Syn in the onset of the disorder^6^,^7^. A very recent study provided evidences for the transfer of Syn from an intrastriatal inoculation to recipient cells, leading to propagation of the pathology along interneuronal circuits^8^. Besides the increase in Syn expression and release due to multiplications of Syn gene, any form of brain injury or cell death during neurodegeneration may promote release of monomeric cellular Syn. In PD models of Syn overexpression, dopaminergic neuron loss is preceded by degenerative changes in striatal axons and terminals, suggesting that Syn-induced pathology hits the axons and terminals first, and cell bodies are involved by a dying back mechanism. Distinct stages of the disease can be mimicked by the time course of alterations occurring in Syn treated animals^9^.

However, the early deficits in neuronal development and functionality elicited by the presence of high levels of extracellular Syn have not been investigated yet.

Adult neurogenesis is affected in ageing brain and in neurodegenerative diseases^10^,^11^, increasing the severity of the pathology due to neuronal loss. A decrease in hippocampal neurogenesis has been found in both PD patients and PD animal models^12^,^13^,^14^. Axon elongation and guidance are fundamental processes for correct migration, integration and connectivity of developing neurons, processes that are finely tuned by actin turnover and actin binding proteins activity.

Interaction between Syn and actin was suggested by a co-localization observed in two neuronal cell lines^15^ and by the dysregulation of actin levels observed in *D. melanogaster* and in *C. elegans* models of PD^16^,^17^. Syn was shown to directly interact with actin *in vitro* and to affect actin dynamics in living cells and neurons in culture^18^. Extracellular monomeric Syn in high dosage, as the A30P mutant form of Syn, was found to impair actin dynamics through the stabilization of microfilaments mediated by cofilin 1 phosphorylation^19^. An altered balance of cofilin 1 activity due to dysregulation of kinases and phosphatases that control cofilin 1 state of phosphorylation has been associated with neurodegeneration. Increased cofilin 1 inactivation/phosphorylation with age and in Alzheimer disease was found due to inactivation of Slingshot phosphatases 1^20^. Amyloid-beta peptide was shown to both decrease cofilin phosphorylation at low doses, promoting actin rod formation^21^, and to inactivate cofilin through the LIM domain kinase (LIMK) pathway at higher doses, contributing to actin polymerization^22^. Lack of leucine-rich repeat serine/threonine-protein kinase (LRRK), or mutant LRRK unable to bind protein kinase A (PKA), increased PKA-dependent phosphorylation of cofilin, leading to abnormal synaptogenesis and transmission^23^.

Here we show that hippocampal neurons in culture exposed to extracellularly-delivered wt or A30P mutant Syn have defective axon elongation and blunted growth cone turning, while actin turnover is decreased and cofilin 1 activity is altered. All the cytoskeletal-associated defects in axonal development induced by Syns can be prevented by activation of cofilin 1. We propose a mechanism by which the accurate wiring of neural networks, both in developing neurons in the areas of adult neurogenesis, and in mature established connections, is lost at early stages during Syn-induced neurodegeneration. Thereby cofilin 1 regulatory pathways might constitute an effective target for therapy of PD.

## Results

### Actin turnover is slower in neurons incubated with Syns

**Figure 1:**
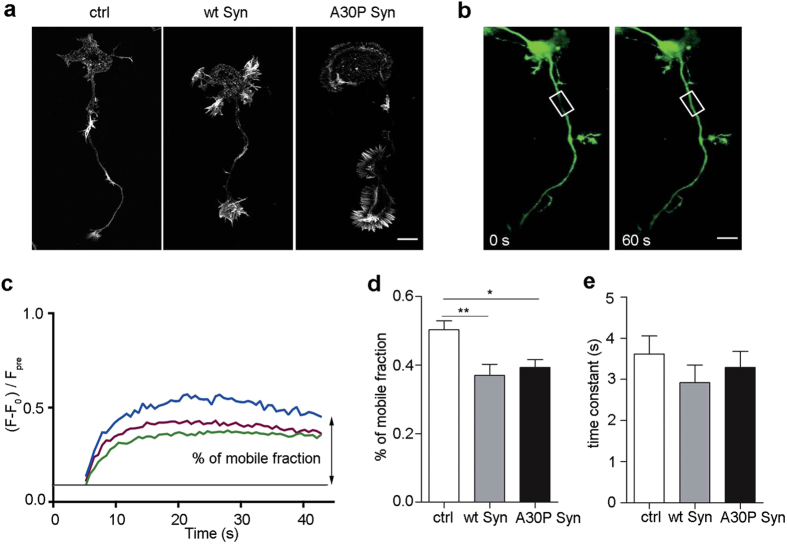
Extracellular Syns decrease actin mobile fraction. (**a**) F-actin distribution, as revealed by fluorescent phalloidin staining, in 2 DIV mouse embryonic hippocampal neurons incubated in the absence or presence of 5 μM purified wt or A30P Syn for 2 days. (**b**) Neurons infected with actin-YFP were bleached along the axon (left panel, square) and imaged at 3 DIV at 1 frame/0.6 s for 60 s (right panel). (**c**) Mean curves of actin-YFP fluorescence recovery after photobleaching over time in control (blue), wt Syn (green) and A30P Syn (red)-treated neurons, notice the lower value of the plateau in the curves obtained from Syns-treated neurons. (**d**) Quantitative evaluation of the percentage of mobile fraction over the total fluorescence and of the time constant of fluorescence recovery (**e**) in control, and in neurons treated with Syns as in (**a)**. In (**d, e**) data are expressed as mean values ± SEM. In (**c**–**e**), ctrl, n = 19, wt Syn, n = 24; A30P Syn, n = 24, from 3 independent cultures. Statistical significance determined by one-way ANOVA followed by Dunnett’s test for multiple comparison, *P < 0.05; **P < 0.01. (**d**) P = 0.0046, alpha value: 0.050:0.791; (**e**) P = 0.5170 n.s., alpha value: 0.050:0.049). Bars in (**a, b)** 10 μm.

We previously showed that Syn physiologically modulates actin assembly, an activity that is profoundly altered in the Syn A30P mutant^18^, and that high levels of soluble extracellular Syns, both wt and A30P mutant form, were also able to affect actin dynamics, stabilizing microfilaments^19^. To assess the chronic effect of Syns present in the extracellular milieu, we incubated embryonic hippocampal neurons immediately after dissection with 5 μM purified human recombinant wt or A30P Syn for 2 days. The purified proteins contained no aggregated or cleaved forms, and showed the presence of few oligomers after 2 days of incubation with the neuronal medium^24^. As compared to control samples, in neurons at 2 days *in vitro* (DIV) exposed to extracellular Syns was evident an increase in filopodia and lamellipodia around the cell body and the growth cone (Fig. 1a), indicating increased actin assembly and stabilization.

To understand whether the observed effect of extracellular Syns on cytoskeleton morphology and dynamics could be due to an increase in stabilized microfilaments, we performed experiments of fluorescence recovery after photobleaching (FRAP). FRAP was performed on neurons at 3 DIV virally infected with actin-YFP. The region of interest (ROI) was chosen in the center of the axon at the same distance from the cell body in all the neurons examined (Fig. 1b). The recovery of fluorescence intensity after photobleaching was quantified after normalization on a control area. The time constant of fluorescence recovery correlates with the velocity of actin polymerization, and the plateau reached by fluorescence indicates the fraction of filaments undergoing active treadmilling (Fig. 1c). Quantification of these parameters showed that in Syns-treated neurons the mobile fraction was decreased by ~35% with respect to control (Fig. 1d), albeit the rate of insertion did not change (Fig. 1e). These results suggest that the pool of free actin monomers is low and the balance is shifted toward a stable, polymerized state of microfilaments.

### Axon turning is hampered in neurons incubated with Syns

**Figure 2:**
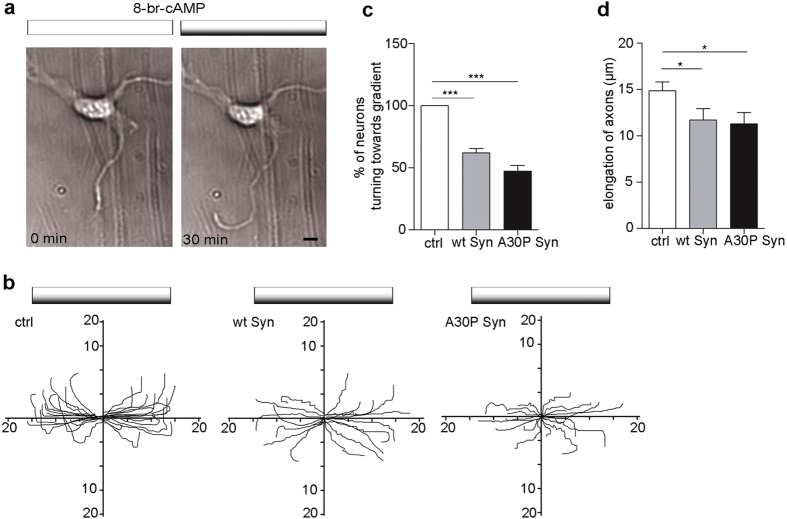
Extracellular Syns hamper axon guidance. (**a**) Neurons cultured on a coverslip assembled upside down over a Dunn’s chamber with the outer well filled with 40 μM cyclic 8-br-cAMP that forms a gradient by diffusion. Neurons were imaged at 1 frame/min for 30 min, images are shown at 0 min and at 30 min from application of the gradient (grey bar on top). (**b**) Trajectory plots of growing axons exposed to a gradient of attractant cue during the 30 min of the experiment in ctrl, wt and A30P Syn-treated neurons at 2 DIV. The source of chemoattractant is at the top of the plots. The initial direction of the axon was aligned with the horizontal axis. (**c**) Quantitative evaluation of the percentage of axons turning toward the attraction gradient in neurons treated as in (**b**). Data from neurons of each experiment were pooled and statistical analysis was performed comparing the data from independent experiments. (**d**) Quantitative evaluation of axon elongation during 30 min in neurons treated with or without wt or A30P Syn. In (**c, d**) data are expressed as mean values ± SEM. In (**c**), ctrl, n = 30; wt Syn, n = 17; A30P Syn, n = 18, from 3 independent cultures. In **d** ctrl, n = 20; wt Syn, n = 14; A30P Syn, n = 12, from 3 independent cultures. Statistical significance was determined by one-way ANOVA followed by Dunnett’s test for multiple comparison, *P < 0.05; ***P < 0.001. (**c**) P = 0.0001, alpha value: 0.050:1.000; (**d**) P = 0.0424, alpha value: 0.050:0.450) Bar in **a**: 10 μm.

Either disruption or stabilization of actin cytoskeleton can cause the inability of the axons to elongate and to turn in response to extracellular cues. The chemotaxis Dunn’s chamber was used to study the response of neurons to a gradient of chemoattractants, the outer well contained a solution of 20 μM 8-Bromoadenosine 3′,5′-cyclic monophosphate (8-Br-cAMP), a PKA activator (Fig. 2a). Neurons were incubated without or with wt and A30P Syn for 2 days prior to the experiments and were examined for their response to 8-br-cAMP by time lapse imaging for 30 min. The analysis of the direction of axon turning in the presence of the attractant cue showed that, while control neurons changed their trajectory of growth in the direction of increasing concentrations of chemoattractant, Syns-treated neurons lost the ability to follow guidance cues (Fig. 2b). Quantification of the direction of growth of the axons after 30 min from application of 8-Br-cAMP indicates 100% positive response in control neurons and a 50% positive response in Syns-treated neurons, which is indicative of a random behavior (Fig. 2c). The growth rate of the turning axons was also significantly decreased in wt and A30P Syn-treated neurons (Fig. 2d). Taken together, these results suggest that the Syns-driven decrease in actin turnover might correlate with functional deficits in developing neurons, such as elongation and correct migration of the axon.

### Actin wave velocity is lower in neurons exposed to extracellular Syns

**Figure 3:**
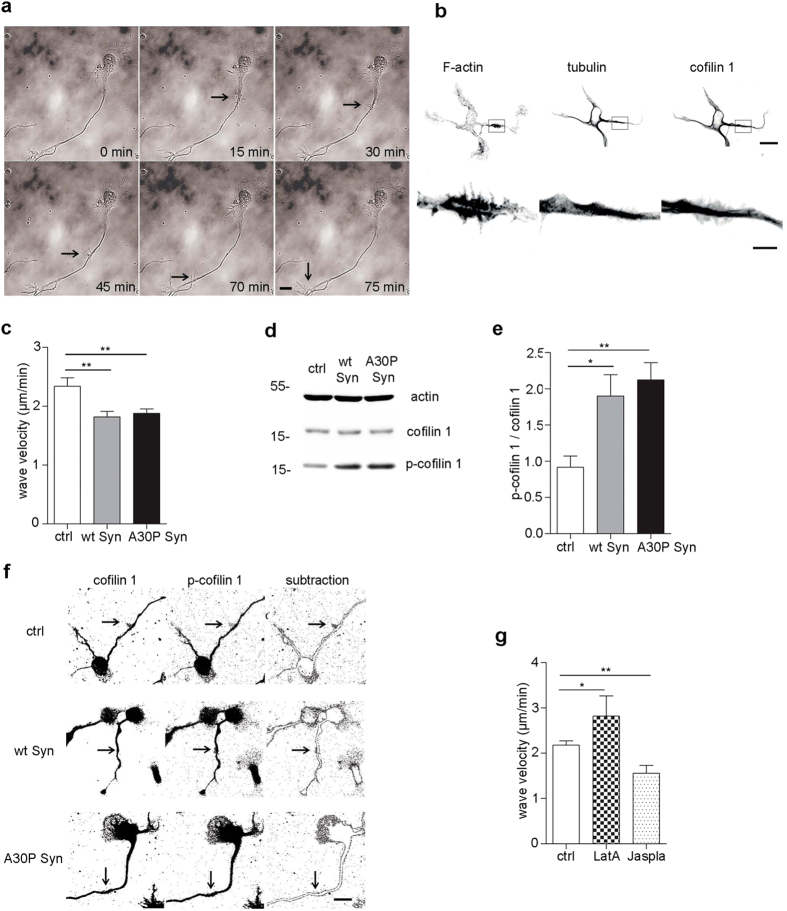
In the presence of Syns actin waves move slower and contain higher concentration of p-cofilin 1. (**a**) Time-lapse images of a 2 DIV embryonic hippocampal neuron showing an actin wave that proceeds from the cell body to the growth cone. Neurons were imaged at 1 frame every 2 min for 3 h. (**b**) Fluorescence analysis of neuron at 2 DIV stained with phalloidin (F-actin) and antibodies to tubulin and cofilin 1. Actin and cofilin 1 are present in the wave (arrows) while tubulin is excluded. (**c**) Quantification of actin waves velocity calculated on neurons treated with or without wt or A30P Syn for 2 days and imaged as in (**a**). (**d**) Representative immunoblots showing levels of proteins indicated on the right in cell homogenates from neurons treated with or without wt or A30P Syn. Actin is shown as an internal standard. (**e**) Quantification of the ratio between p-cofilin 1 and cofilin 1 bands, analyzed by densitometry and normalized on actin level, for each experimental condition shown in (**d**). (**f**) Fluorescence analysis of neurons incubated for 2 days with or without wt or A30P Syn and processed for cofilin 1 and p-cofilin 1. In the right panels are shown images derived from the subtraction of the area stained for p-cofilin 1 from the area stained for cofilin 1. (**g**) Quantification of the velocity of actin waves calculated on neurons treated with or without 2 μM LatA for 20 min or 12 nM Jaspla for 20 min. In (**c, e, g**) data are expressed as mean values ± SEM. In **c**, ctrl, n = 59; wt Syn, n = 60; A30P Syn, n = 76, from 5 independent cultures. In (**e**), n = 3 independent experiments. In (**g**), ctrl, n = 74; LatA, n = 20; Jaspla, n = 23, from 3 independent cultures. Statistical significance was determined by one-way ANOVA followed by Dunnett’s test for multiple comparison, *P < 0.05; **P < 0.01. (**c**) P = 0.0012, alpha value: 0.050:0.890; (**e**) P = 0.0086, alpha value: 0.050:0.477; (**g**) P = 0.0012, alpha value: 0.050:0.897). Bars in (**a, f**) 10 μm, in (**b**) upper row, 10 μm, lower row, 2 μm.

In growing neurons actin waves travel from the cell body to the growth cone to facilitate the growth of the axon. We used the velocity of actin waves as a parameter of neurite dynamicity; indeed actin waves are more frequent and faster in neurons at early DIV. During time-lapse imaging of neurons at 2 DIV, growth cone-like structures travelling from the cell body to the tip of the axon were clearly visible (Fig. 3a). Actin waves contained actin and cofilin 1, an actin binding protein, while microtubules, which are present in the center of the axon, were absent from actin waves (Fig. 3b). Neurons incubated with or without Syns were imaged at 1 frame/2 min and the velocity of actin waves was determined calculating the time taken by the wave to reach the tip of the growth cone, normalized for the length of the axon (Supplementary Video 1). As compared to control samples, neurons exposed to extracellular Syns for 2 days showed a ~20% decrease in the velocity of actin waves (Fig. 3c), even though they looked healthy and exhibited dynamic growth cones. We previously showed that extracellular Syns indirectly act on actin dynamics through inactivation of cofilin 1, promoting stabilization of actin filaments^19^. Thus we wondered if cofilin 1 was in its inactive state after chronic exposure to Syns. Neurons were exposed to Syns for 2 days and the level of phosphorylated, inactive cofilin 1, was quantified by immunoblot (Fig. 3d). The level of phosphorylated cofilin 1 was indeed increased 1.8 fold in neurons incubated with wt Syn and 2 fold in neurons incubated with A30P Syn compared to control (Fig. 3e). The study of the localization of inactive cofilin 1 was performed subtracting phospho-cofilin 1 immunofluorescent areas from the areas stained by fluorescent cofilin 1 in neurons treated or not with Syns for 2 days (Fig. 3f). Subtraction images revealed areas of enriched phospho-cofilin 1 where the staining was absent, and in particular actin waves showed no staining in Syns-treated neurons (Fig. 3f, middle and lower panels, arrows), suggesting that, through inactivation of cofilin 1, actin cytoskeleton lost its dynamics inside actin waves. To further assess the correlation between actin waves velocity and cytoskeletal polymerization state, we used drugs that modulate actin assembly. Neurons were exposed to Jasplakinolide (Jaspla), a stabilizer of microfilaments, and to Latrunculin A (LatA), a monomers sequestering protein, for 20 min, the drugs were washed out and the neurons were imaged for 3 h. Indeed, after the treatment with Jaspla, actin waves velocity was reduced by 20%, similarly to Syn effect, and upon LatA treatment actin waves velocity was higher (Fig. 3g), suggesting that depolymerization bursts the movement of actin waves.

### The velocity of actin waves is reduced after axonal injury and Syns favor the healing process

**Figure 4:**
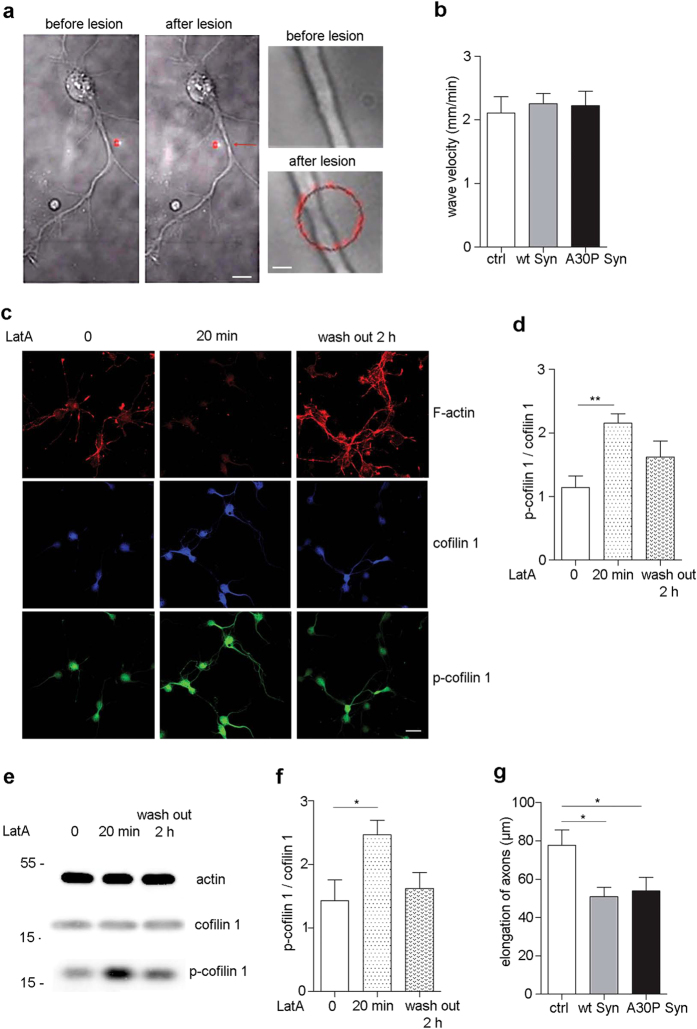
Syns favor the movement of actin waves after axonal injury. (**a**) Neuron at 2 DIV before (left panel) and after (middle panel) injury by UVA laser (red spot). In the panels on the right is showed a magnification of the lesion area before (upper) and after (lower) injury. (**b**) Quantification of the velocity of actin waves in injured neurons treated with or without wt or A30P Syn for 2 days and imaged as in Fig.3a. (**c**) Fluorescence analysis (Z-stack of confocal images) of embryonic hippocampal neurons before (left panels), after 20 min of 2 μM LatA (middle panels), and after 2 h of wash out of the drug (right panels), and stained with phalloidin (F-actin) (red), and antibodies to cofilin 1 (blue) and p-cofilin 1 (green). (**d**) Quantification of the ratio between the fluorescence intensity of p-cofilin 1 and cofilin 1, for each experimental condition shown in (**c**). (**e**) Representative immunoblots showing levels of proteins indicated on the right in cell homogenates from neurons treated with or without 2 μM LatA for 20 min and after 2 h of wash out of the drug. Actin is shown as an internal standard. (**f**) Quantification of the ratio between p-cofilin 1 and cofilin 1 bands, analyzed by densitometry, for each experimental condition shown in **e**. (**g**) Quantification of axon elongation during 16 h in neurons treated with or without wt or A30P Syn for 2 days and injured by UVA laser. In (**b, d, f, g**) data are expressed as mean values ± SEM. In (**b**), ctrl, n = 30; wt Syn, n = 35; A30P Syn, n = 32, from 3 independent cultures. In (**d**), n = 5 for each condition from 3 independent cultures. In (**f**), n = 3 independent experiments. In (**g**), ctrl, n = 25; wt Syn, n = 18; A30P Syn, n = 13, from 4 independent cultures. Statistical significance was determined by one-way ANOVA followed by Dunnett’s test for multiple comparison, *P < 0.05; **P < 0.01. (**b**) P = 0.2956, n.s., alpha value: 0.050:0.083; (**d**) P = 0.0043, alpha value: 0.050:0.942; (**f**) P = 0.0316, alpha value: 0.050:0.683; (**g**) P = 0.0141, alpha value: 0.050:0.659). Bars in (**a**) middle panel, 10 μm, right panel, 1 μm, in (**c**) 20 μm.

Brain trauma and lesion of neurons is a condition that deepens PD symptoms. Actin cytoskeleton is involved in repair and regeneration of injured axons, therefore we wondered if Syns would interfere with the healing process. A sub-nanosecond pulsed UVA laser was used to create a partial lesion of axons in a highly controlled and reproducible manner. The low power of the laser (1.8 μW) induced thinning of the axon without complete ablation of the neuronal membrane (Fig. 4a, red spot, and Supplementary Video 2), and a localized disruption of the three elements of the axonal cytoskeleton^25^. The axon and the growth cone that shrunk after lesion returned to the original shape in short time, and in few min actin waves started moving over the lesion proceeding to the growth cone (Supplementary Video 3). The wave velocity at steady state in the absence or presence of Syns was used to compare dynamics in injured and uninjured neurons.

In control neurons, the lesion induced a slight decrease of the velocity of actin waves. Unexpectedly, in wt and A30P Syn-treated neurons the velocity of actin waves after lesion was similar to control (Fig. 4b), however significantly higher in respect to the velocity of actin waves in Syns-treated, not lesioned neurons (Fig. 3c). It is conceivable that, in a condition in which actin becomes resistant to depolymerization and microfilaments are stabilized, as is in the presence of Syns, the pool of actin monomers needed for active filaments treadmilling is poor, leading to slow movement of actin waves. In case of local disruption of microfilaments, as after injury, the pool of monomers is locally restored favoring active polymerization.

In order to verify this hypothesis we treated neurons with LatA. LatA, inducing actin depolymerization, might represent a condition mimicking lesion, albeit not localized, but involving the whole axon. We showed that the velocity of actin waves was higher upon LatA treatment (Fig. 3g), and we asked which might be the common target for Syns and LatA that can favor the healing process. A generalized depolymerization, as is induced by LatA, conceivably might activate a feedback mechanism in the cells, aimed at the rescue of microfilaments morphology. When LatA was applied to neurons for 20 min it was observed an increase in the level of phosphorylated cofilin 1, that returned to control conditions after 2 h from wash out of the drug, evident in the immunostaining (Fig. 4c,d) and in the immunoblot of p-cofilin 1 (Fig. 4e,f). The inactivation of cofilin 1, which will favor polymerization of microfilaments, would explain the increase in the velocity of actin waves in the presence of the drug, and, similarly, in the presence of Syns after microfilaments have been depolymerized by the laser-induced injury. The acute protective effect of Syns due to inactivation of cofilin 1, did not exclude a possible alteration in axon elongation after injury at later time points. Neurons were incubated with Syns after plating, at 2 DIV the axon was injured as above, and neurons were imaged for 16 h. The axon was traced and the increase in length was calculated between the first and the last frame of the movie (Fig. 4g). Indeed, after injury and in the presence of Syns, elongation was 30% less than in control, indicating that although the chronic inactivation of cofilin 1 could transiently favor re-polymerization of disrupted filaments, it impairs regeneration of the axon at later time points.

### Activation of cofilin 1 prevents the effects of extracellular Syns

**Figure 5:**
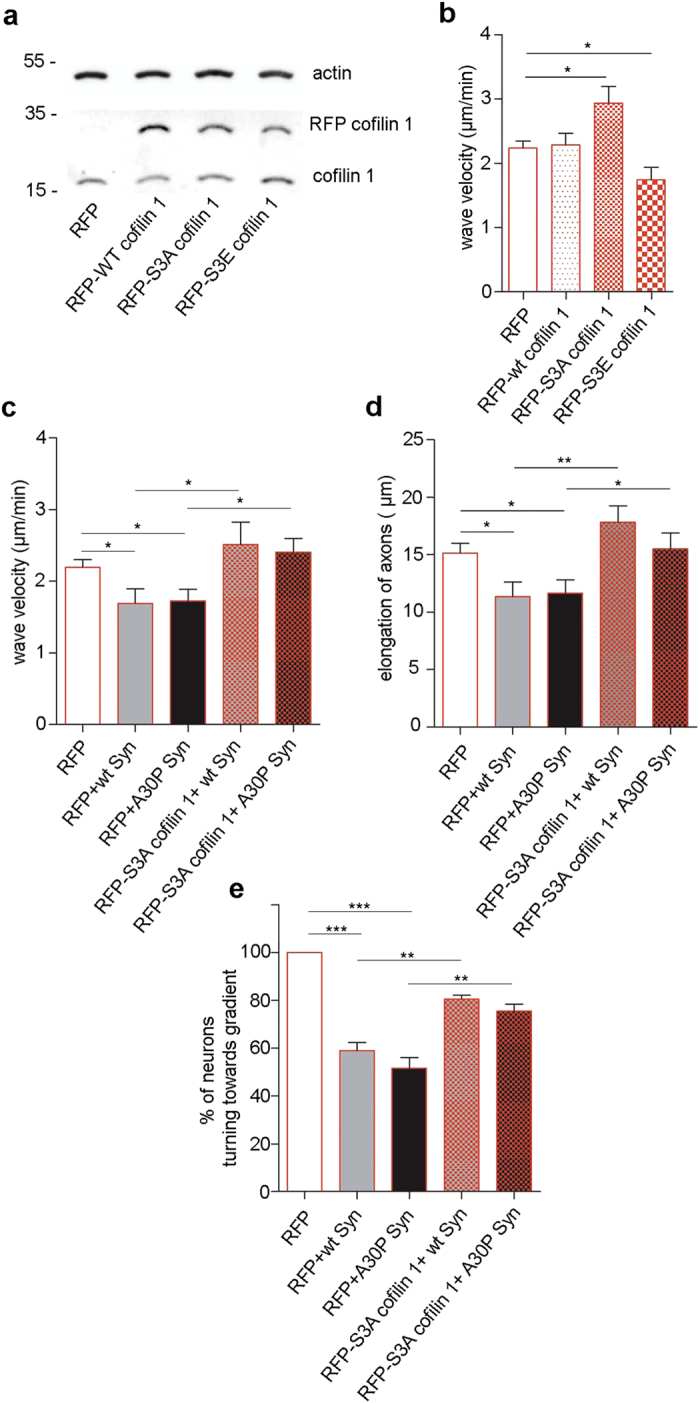
Active cofilin 1 prevents Syn-driven defects in axon elongation and turning. (**a**) Immunoblots showing the proteins indicated on the right in homogenates from neurons electroporated with RFP-wt cofilin, or RFP-S3A cofilin, or RFP-S3E cofilin, or RFP as a control. (**b**) Quantification of the velocity of actin waves in neurons electroporated with constructs as in (**a**). (**c**) Quantification of the velocity of actin waves in neurons electroporated with RFP or RFP-S3A cofilin and incubated in the absence or presence of wt or A30P Syn for 2 days. (**d**) Quantification of axon elongation during 30 min in neurons electroporated with RFP or RFP-S3A cofilin and treated with or without wt or A30P Syn. (**e**) Quantification of the percentage of axons turning toward the attraction gradient in neurons electroporated and treated as in (**d**). Data from neurons of each experiment were pooled and statistical analysis was performed comparing the data from independent experiments. In (**b**–**e)** data are expressed as mean values ± SEM. Statistical significance was determined by two tailed t-test. In (**b**), RFP/S3A, P = 0.0176, alpha value: 0.050:0.747; RFP/S3E, P = 0.0381, alpha value: 0.050:0.273; RFP, n = 41; wt cofilin, n = 13; S3A, n = 43; S3E, n = 11, from 3 independent cultures. In **c**, RFP/wt Syn, P = 0.0226, alpha value: 0.050:0.547; RFP/A30P Syn P = 0.0191, alpha value: 0.050:0.181; wt Syn/S3A + wt Syn P = 0.0291, alpha value: 0.050:0.604; A30P Syn/S3A + A30P Syn, P = 0.0135, alpha value: 0.050:0.421; RFP, n = 43; wt Syn, n = 17; A30P Syn, n = 20; S3A + wt Syn, n = 14; S3A + A30P Syn, n = 27, from 3 independent cultures. In **d**, RFP/wt Syn P = 0.0231, alpha value: 0.050:0.634; RFP/A30P Syn P = 0.0282, alpha value: 0.050:0.602; wt Syn/S3A + wt Syn, P = 0.0035, alpha value: 0.050:0.884; A30P Syn/S3A + A30P Syn, P = 0.0426, alpha value: 0.050:0.540; RFP, n = 33; wt Syn, n = 13; A30P Syn, n = 13; S3A + wt Syn, n = 9; S3A + A30P Syn, n = 10, from 3 independent cultures. In **e**, statistical significance was determined by one-way ANOVA followed by Dunnett’s test for multiple comparison, ***P < 0.001, (RFP/wt Syn, RFP/A30P Syn, P = 0.0001, alpha value: 0.050:1.000), and by two tailed t-test: wt Syn/S3A + wt Syn P = 0.0012, alpha value: 0.050:0.874; A30P Syn/S3A + A30P Syn, P = 0.0044, alpha value: 0.050:0.688; RFP, n = 43; wt Syn, n = 17; A30P Syn, n = 20; S3A + wt Syn, n = 14; S3A + A30P Syn, n = 27, from 3 independent cultures.

To correlate cofilin 1 phosphorylation state, and therefore cytoskeleton stabilization, with Syn-driven decrease of the velocity of actin waves, we used dominant negative and positive cofilin 1 constructs. Neurons were electroporated with either wild-type cofilin 1, or the non-phosphorylatable S3A mutant form of cofilin 1, which is constitutively active, or the pseudophosphorylated S3E mutant, which is constitutively inactive. Neurons electroporated with RFP tagged constructs of cofilin 1 expressed the exogenous protein (Fig. 5a, RFP-cofilin) at an average 2 to 4 fold the level of endogenous cofilin 1, as calculated considering the transfection efficiency, which was ~30%–40%. S3A cofilin 1 overexpression induced significantly faster waves and the opposite was true in S3E cofilin 1 overexpressing neurons (Fig. 5b), indicating the importance of cofilin 1 modulation to promote actin wave motility. Importantly, overexpression of S3A cofilin 1 in neurons exposed to Syns was able to restore the rate of movement of actin waves (Fig. 5c), balancing Syn-driven inactivation of the protein.

Based on these results, and to correlate the rate of movement of actin waves with axonal elongation, we tested the ability of constitutively active cofilin 1 to prevent the defects in axonal growth rate. Neurons electroporated with S3A cofilin 1 and exposed for 2 days to Syns as above, were imaged, and the length of the axon was calculated after 30 min. Exposure of mock-transfected neurons to wt and A30P Syn induced a significant decrease in elongation of axons as was observed in non transfected neurons (Fig. 2d), the expression of active cofilin 1 was able to restore the physiological growth rate in the presence of Syns (Fig. 5d).

Further, to confirm the correlation between the effect of Syn on cofilin activity, the resulting alteration of cytoskeleton dynamics, and growth cone turning, we assessed the capability of active cofilin 1 to restore the response of the axon to attractant cues, which was lost in the presence of extracellular Syns. Neurons mock-transfected or expressing S3A cofilin 1 were analyzed in the Dunn’s chamber upon creation of a gradient of 8-Br-cAMP. While only 50% of RFP expressing neurons incubated with Syns randomly turned toward the attractant cue, the expression of S3A cofilin 1 reinstated the correct response and 80–100% of axons were able to turn following the gradient (Fig. 5e).

Taken together these results indicate the possibility to prevent Syn effect acting on its target protein cofilin 1, and to reinstate the capability of neurons to elongate and to migrate toward their target.

## Discussion

In this study we provide evidence that extracellularly released Syn contributes to the deficits in axon elongation and guidance that underlie the impairment in neuronal development and regeneration after injury, participating in the severity of neurodegenerative diseases, such as PD.

We previously demonstrated that actin dynamics are affected by the presence of excess wt or A30P Syn in the extracellular milieu, resulting in microfilament stabilization^19^. Here, we show that long-term exposure of neurons to extracellular Syns correlates with a decreased actin turnover, indicated by the low percentage of mobile actin along the axon. Neurons appeared enriched in actin structures, with large lamellipodia around the cell body and at the neurite tip, reminiscent of the effect induced by actin stabilizing drugs.

Multiplications of Syn-coding gene occur in early onset PD^2^,^26^,^27^, and, consistently, high concentrations of wt Syn were acting similarly to A30P mutated Syn, confirming the pathological role of an excess of the wt protein. Interestingly, the selective vulnerability of dopaminergic neurons in the substantia nigra in respect to the neurons of the ventral tegmental area may correlate to the increased Syn levels found in these neurons in aged monkeys and humans^28^.

Axonal injury, an underlying cause of neurodegenerative disorders, triggers changes in gene expression, proteins complexes and proteins re-localization. Syn enrichment in damaged axons^29^ suggests a possible role in regenerative sprouting and growth cone pathfinding. Actin dynamics are necessary for axon guidance and unguided axon growth occurs in the absence of actin assembly^30^. Regional disruption of actin induces the growth cone to turn away from this region and to grow in the direction containing stabilized filopodia^31^.

We report that chronic exposure to extracellular Syns slows axonal elongation both during physiological growth and during re-growth after lesion. We found that the velocity of actin waves, used as a parameter of neurite dynamicity^32^, correlated with axon extension, and was decreased in the presence of Syns. Actin waves were shown to contain actin and cofilin 1, while Syns treatment induced accumulation of phosphorylated cofilin 1 in actin waves. Consistently, the velocity of actin waves was increased in the presence of the actin depolymerising drug, LatA, and decreased in the presence of the actin stabilizing drug, Jaspla. After axonal injury, microfilaments were partially interrupted, as was shown in our previous results^25^, and a shift of actin dynamics toward polymerization favoured healing of the lesion, in fact the velocity of actin waves in the presence of Syns was higher than before the lesion. However, at later time points, regeneration of the axon was hampered by the decreased actin turnover due to Syn presence. The correlation between cofilin 1 activity and the velocity of actin waves was assessed with the use of LatA. In a condition of microfilament depolymerization induced by LatA, similarly to the situation in the injured axon, the velocity of actin waves increased and cofilin 1 was inactivated as a feedback mechanism. After washout of LatA, cofilin 1 was dephosphorylated to control condition, restoring its activity to balance actin dynamics, while in the presence of Syns, cofilin 1 remained chronically inactive.

Cofilin 1, an actin severing protein, maintains the pool of actin monomers and thus remodels actin filaments by enhancing assembly/disassembly dynamics^33^. Phosphorylation of cofilin 1 at a conserved serine3 residue by multiple kinases, including LIMK, leads to inhibition of its actin depolymerizing activity^34^,^35^. Cofilin 1 activity is involved in axon extension and growth cone motility^36^,^37^,^38^, and in retinal neurons cofilin 1 acts as a target of brain-derived neurotrophic factor (BDNF) in the modulation of filopodial dynamics^39^,^40^.

Moreover, a role for cofilin in growth cone guidance was recently shown in response to bone morphogenic proteins in X*enopus laevis* spinal neurons^41^, and nerve growth factor (NGF) and netrin-1 were shown to stimulate plasma membrane protrusion in growth cone, while increasing active cofilin. Local increases in active cofilin induced attractive turning, and reduced cofilin activity blunted turning to NGF or netrin-1^42^.

We previously showed that Syns induced cofilin 1 inactivation through the Rac1/LIMK pathway triggered by the accumulation of glucose-related protein of 78 kDa (GRP78) at the external surface of the plasma membrane. Here we provide evidence that Syn, present for 2 days in the neuronal medium, maintained a chronic phosphorylation of cofilin 1, and that in these neurons the response of the growth cone to attractant cues was lost. Indeed, the expression of active cofilin 1 in neurons was able to restore attractive turning of the growth cone. Consistently, expression of active cofilin 1 was also preventing the deficits in axon elongation and in the rate of movement of actin waves induced by Syns, indicating cofilin 1 as a main target of Syns pathological activity during development of newborn neurons and regeneration of lesioned neurons. Increased cell death and reduced survival of newborn neurons in Syn transgenic animals may be due to defects in outgrowth and synaptic integration and it has been shown the toxicity of increasing amount of Syn to exert these pathological effects^12^.

Moreover, the connection between cofilin 1 activity and neurotrophins, which were also shown to restore the number and the rate of movement of actin waves^25^, reinforces the use of neurotrophins as a therapeutic target in the treatment of PD and related synucleopathies.

## Materials and Methods

### Reagents

All chemical reagents were purchased from Sigma Aldrich (St. Louis, MO) and GE Healthcare (Uppsala, Sweden). Glass bottom Petri dishes were from Mattek Corp (Ashland, MA) and Dunn’s chamber was from Hawksley (Lancing, UK).

### Antibodies and fluorescent probes

Mouse monoclonal antibodies: anti-actin (Sigma Aldrich); anti-cofilin 1 (Abcam, Cambridge, MA). Rabbit polyclonal antibodies: anti-phosphorylated cofilin 1 (Santa Cruz Biotechnology, Santa Cruz, CA); anti-b-tubulin III (Covance, Emervylle, CA).

Fluorescent-conjugated Alexa Fluor secondary antibodies were from Molecular Probes (Eugene, OR); horseradish peroxidase-conjugated secondary antibodies (Bio-Rad, Hercules, CA); FITC-conjugated phalloidin (Molecular Probes-Life Technologies, Carlsbad, CA).

### Plasmids

DNAs of pmRFP-N1 human cofilin wt (catalogue number 50856), pmRFP-N1 human cofilin S3A (catalogue number 50857) and pmRFP-N1 human cofilin S3E (catalogue number 50858) (Addgene (Cambridge, MA) were a gift from James Bamburg.

### Protein purification

Constructs encoding the human full-length wt or A30P Syn inserted in the pET21d plasmid were a kind gift from Dr. Brett Lauring (Columbia University, New York, NY, USA). Bacteria were induced during the exponential phase with 1 mM isopropyl-D-1- thiogalactopyranoside for 2 h and harvested by centrifugation. The pellet was solubilized in Buffer A (HEPES/KOH 20 mM, pH 7.2 and KCl 100 mM) and heated for 5 min at 90 °C. The cell lysate was centrifuged at 72,000 × g for 30 min, and the supernatant was loaded on a HiTrap monoQ column Syn and was eluted with a liner gradient of KCl from 100 to 500 mM, and the fractions of interest were concentrated using a Centricon centrifugal filter before loading on a Superose 12 column in Buffer A. The fractions containing Syn were pooled, concentrated and stored at −80 °C. To control Syn purity, 1 mg of purified Syn was loaded on top of a Superdex 75 10/300 column (GE Healthcare) and eluted at 0.5 ml/min on an AKTA Purifier apparatus. Optical density (OD) was continuously measured at 280 nm, and 50 ng of purified Syn were loaded on an SDS-PAGE gel and stained with Coomassie Brilliant Blue.

### Primary embryonic neuronal cultures

Primary neuronal cultures were obtained from hippocampi dissected from C57BL/6S E 18 mice (Harlan, Udine, Italy). Animals were maintained in a pathogen free animal facility. All experiments were performed in strict accordance with experimental procedures approved by the Italian Ministry of Health.

Embryos were removed and dissected under sterile conditions. Hippocampi were dissociated by enzymatic digestion in trypsin (0.125% for 30 min at 37 °C). Trypsin activity was blocked by adding complete media (NeurobasalTM (Gibco) supplemented by B27 (2%, Gibco), alanyl- glutamine (2 mM, Gibco), penicillin/streptomycin (both 1 mM, Sigma) containing 10% fetal bovine serum (FBS, Gibco). After trypsinization, tissues were rinsed in complete media without FBS, and dissociated with a plastic pipette. Neurons were plated either on glass-bottom Petri dishes for long-term imaging (density of 20,000 neurons on 10 mm diameter glass bottom); or on glass coverslips for immunocytochemistry (density of 30,000 neurons on 18 mm diameter coverslips); or on glass coverslips (square #3D) for chemotaxy assay (density of 50,000 neurons on 20 × 26 mm coverslip); or on plastic Petri multi wells dishes for immunoblot (density of 350,000 neurons on 30 mm diameter dish). After 2 h from plating, 2 ml serum-free glial conditioned medium was added.

### Glia culture and conditioned medium

Cortices were dissected from E18 mice and put in cold HBSS, and then incubated in trypsin 0.125% + 1 mg/ml of DNAse for 15 min at room temperature, the supernatant was stored and the incubation in trypsin repeated. Two supernatants were pooled and centrifuged for 5 min at 1500 rpm at 8 °C. The cells were resuspended in MEM + 10% Horse serum, Pen/Strep, 33 mM Glucose and 2 mM Glutamine. Glial cells were plated on T75 flasks coated with 0.01 mg/ml Poly-D-Lysine. When 100% confluence was reached, MEM medium was substituted with Neurobasal complete medium. After 2 days the glial conditioned medium was added to hippocampal neurons.

### Electroporation of hippocampal neurons

Primary hippocampal neurons were electroporated in suspension immediately after dissociation, using the Basic Nucleofector Kit for primary neurons on the O-05 program (Amaxa Biosystems, Cologne, Germany). Neurons were electroporated with the constructs of interest (800 ng pmRFP-N1 human cofilin wt, 400 ng pmRFP-N1 human cofilin S3A, 400 ng pmRFP-N1 human cofilin S3E/1,000,000 neurons) at the 4D-NucleofectorTM Core Unit (Amaxa), and plated on glass coverslips, or on plastic Petri dishes, as described above. Electroporated neurons were used at 3 DIV.

### Protein extraction and lysis

DIV neurons were washed with ice-cold HBSS on ice and scraped in Lysis Buffer (100 μl of 1% SDS). Samples were boiled for 2 min at 100 °C, sonicated for 5 s, and centrifuged for 15 min at 12,000 rpm. 5× sample buffer (250 mM TrisHCl pH6.8, 10% SDS, 30% Glycerol 30, 5% β-mercapitalethanol, 0.02% bromophenol blue) was added to each protein sample. Proteins were separated by SDS polyacrylamide gel electrophoresis (PAGE) and immunoblotting.

### Immunocytochemistry

Hippocampal neurons were plated and treated with wt or A30P Syn for 2 days; control incubations were obtained by adding the chromatographic elution buffer. Neurons were fixed with 4% PFA and stained with FITC-conjugated phalloidin for 1 hour. Neurons treated with 2 μM LatA (Molecular Probes-Life Technologies) were fixed at 20 min, and at 2 h after Lat A washout. Neurons treated with 12 nM Jaspla (Molecular Probes-Life Technologies) were fixed at 20 min. Confocal images were acquired in a Leica SP2 confocal microscope, using a Leica 63× objective lens (NA 1.4; Leica Microsystems, Wetzlar, Germany). All images were analyzed with ImageJ (NIH, Bethesda, MD).

### Time Lapse microscopy

Long-term bright field time-lapse experiments (up to 16 h) were performed on a commercial inverted microscope (Eclipse Ti E; Nikon Instruments Inc.) with laser-based autofocus and motorized stage for multipoint sequential image acquisition. The microscope was equipped with a charge-coupled device (CCD) camera (Andor DU-897D-C00), Plan Fluor 40×, 0.75 NA DIC objective and imaging software (Nis elements AR; Nikon Instruments Inc.) Around 10 neurons were imaged for each condition and images were taken with a frame interval of 2 min.

For long-term time-lapse imaging, neurons were kept at a stable temperature of 35 °C in a custom-built cell incubator stage adaptable for inverted microscope^43^. To maintain the physiological pH, a gas of 5% CO_2_ balanced with air was mixed by a flow meter (CO2BX, Okolab) and passed in the sample holder. Moreover, a film of oil (polydimethylsiloxane 200 Fluid, 0.913 g/ml, Sigma Aldrich), permeable to CO_2_ but not to water, was deposited on the culture media. Images of neurons were selected by multipoint acquisition and imaged at the interval of 1 or 2 min per frame.

### Laser Dissector system for axonal injury

The laser dissection source was a pulsed sub-nanosecond UV Nd:YAG laser at 355 nm (PNV001525-040, PowerChipnano-Pulse UV laser, Teem Photonics, Meylan, France). The damage to the axon appeared restricted to the formation of a neck when the pulse energy was reduced to 1.8 μW. An ablation was made in the middle of the axon^44^.

### Assembling of the Dunn’s Chamber and axon guidance by chemotaxis

The Dunn’s chambers were pre-washed with Neurobasal and then twice with glia conditioned media. Conditioned media was added to fill the inner and outer wells. The coverslip containing the neurons was inverted over the Dunn’s chamber, leaving a narrow slit at the edge for draining and refilling the outer well. Excess media was removed by blotting with filter paper, and three sides of the Dunn’s chamber were sealed with hot paraffin:vaseline (1:1). Using a gel-loading tip, all the liquid from the outer well was removed through the filling slit, and 40 μM 8-br-cAMP (BioLog Life Science Institute, Bremen, Germany) diluted in conditioned media was added to the outer well. The filling slit was then sealed with hot paraffin:vaseline. Dunn’s chamber was assembled rapidly to avoid changes in the pH of the media. After assembly of the Dunn’s chamber, imaging was started immediately. Time-lapse phase contrast images were acquired every min, for 30 min, using a 20× objective. For each Dunn’s chamber, ~15 stage positions were imaged at various locations around the annular bridge. The % of neurons that turned toward the gradient was then calculated.

### Actin turnover analyzed by FRAP

Hippocampal neurons plated on glass-bottom Petri dishes filled with 2 ml serum-free glial conditioned medium, were infected with pLenti4-actin YFP virus at DIV 1. Neurons were incubated in the presence or absence of 5 μM wt or A30P Syn for 2 days. Experiments were performed with neurons at 3 DIV. FRAP experiments were conducted on Leica TCS SP5 microscope using a 63× oil objective (Leica Microsystems). Region of interest (ROI) were selected and the emission signal acquired with 488 nm laser at 20% of intensity, as a pre-bleaching signal. The ROI was subsequently photobleached by three lasers at 458 nm, 476 nm and 488 nm, all set at 100% of intensity. The post-bleaching acquisition was carried out for 60 frames at a rate of 1 frame/0.6 s. The intensity of the ROI for each frame was normalized to the average initial intensity of pre-bleaching frames, and to the area of the bleached axon. The recovery of fluorescence was corrected for the unwanted photobleaching occurring during live imaging quantified in a control region in the field of view. The intensity of the florescence (F) at every time point was automatically generated by Leica Analyzing Software. The average intensity calculated in the first 5 frames corresponds to the pre-bleach (Fpre) value, the average intensity of the 8^th^ frame is the intensity value (F0) after the photobleaching phase occurring in the 6^th^ and 7^th^ frame.

Individual plots of each experiment were fitted to a model function. The fluorescence recovery of actin-YFP was best fitted by a single-exponential function (a double exponential does not significantly improved the quality of the fit), revealing the presence of mobile actin fractions with different recovery kinetics. The following model was applied:


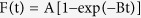


- A is the % of mobile fraction of actin.

- B is the time constant of fluorescence recovery.

A and B were obtained from the fitting of each curve. The mean ± SEM was calculated for the % of recovery of fluorescence, and for the time constant of fluorescence recovery, for each condition.

The final graph shows the normalized fluorescence intensity value of (F − F0)/Fpre on the Y axis and time (s) on the X axis. Fitting of the curve was done in Matlab. Statistical analysis was performed on GraphPad software.

### Image elaboration and statistics

Images composition and drawing were done with the use of Adobe Photoshop (Adobe System, San Jose, CA). Data were analyzed using GraphPad Prism software (Graph Pad, La Jolla, CA), and expressed as mean values ± standard error (SEM). Statistical significance was determined by one-way ANOVA followed by Dunnett’s test for multiple comparisons, (P values < 0.05 were considered significant).

## Additional Information

**How to cite this article**: Tilve, S. *et al.* Cofilin 1 activation prevents the defects in axon elongation and guidance induced by extracellular alpha-synuclein. *Sci. Rep.* **5**, 16524; doi: 10.1038/srep16524 (2015).

## Supplementary Material

Supplementary Video 1

Supplementary Video 2

Supplementary Video 3

Supplementary Video Legends
