# Supplementary material for: Cofilin 1 activation prevents the defects in axon elongation and guidance induced by extracellular alpha-synuclein
Source: Sci Rep. 2015 Nov 12;5:16524. doi: 10.1038/srep16524 (PMC4642265; doi:10.1038/srep16524)
Supplement: Supplementary Video Legends [file srep16524-s4.pdf]

Cofilin 1 activation prevents the defects in axon elongation and guidance induced by extracellular alpha-synuclein

Sharada Tilve, Francesco Difato* and Evelina Chieregatti*
Department of Neuroscience and Brain Technologies, Istituto Italiano di Tecnologia, 16163 Genoa, Italy

Video legends

Video 1. Actin waves movement along the axon. Time-lapse movie of hippocampal neuron at 2 DIV showing actin waves that travel toward the growth cone. Images were taken with a 40x objective at a rate of 10 frames per s.

Video 2. Lesion of the axon by laser. Time-lapse movie of hippocampal neuron at 2 DIV injured by UVA laser showing thinning of the axon without severing of the membrane. Images were taken with a 60x objective at a rate of 3 frames per s.

Video 3. Actin waves after injury. Time-lapse movie of hippocampal neuron at 2 DIV immediately after injury (shown in Video 2), showing healing and movement of actin waves. The arrow indicates the lesion area. Images were taken with a 40x objective at a rate of 10 frames per s.
